# 1-Benzyl-1*H*-benzimidazole

**DOI:** 10.1107/S1600536809039051

**Published:** 2009-10-03

**Authors:** Gang Lei, Li Zhou

**Affiliations:** a School of Chemistry and Chemical Engineering, China West Normal University, Nanchong 637002, People’s Republic of China

## Abstract

In the title mol­ecule, C_14_H_12_N_2_, the benzimidazole ring system is essentially planar (r.m.s. deviation = 0.024 Å). The dihedral angle between the imidazole ring and the benzyl ring is 85.77 (4)°. In the crystal, mol­ecules are linked into chains along the *a* axis by C—H⋯N hydrogen bonds. In addition, the packing is stabilized by C—H⋯π inter­actions involving both six-membered rings.

## Related literature

For general background to benzimidazole derivatives, see: Ansari & Lal (2009 ▶). For the synthesis, see: Hayat *et al.* (2001 ▶).
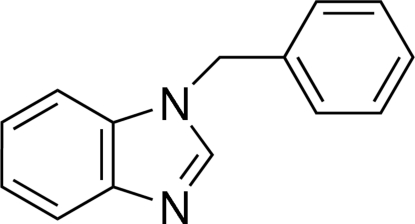


## Experimental

### 

#### Crystal data

C_14_H_12_N_2_
*M* *_r_* = 208.26
Monoclinic, 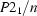

*a* = 6.2265 (10) Å
*b* = 8.1740 (13) Å
*c* = 20.975 (4) Å
β = 97.839 (2)°
*V* = 1057.5 (3) Å^3^
*Z* = 4
Mo *K*α radiation
μ = 0.08 mm^−1^
*T* = 93 K
0.57 × 0.50 × 0.37 mm

#### Data collection

Rigaku SPIDER diffractometer
Absorption correction: none
8358 measured reflections
2412 independent reflections
2198 reflections with *I* > 2σ(*I*)
*R*_int_ = 0.022

#### Refinement

*R*[*F* ^2^ > 2σ(*F* ^2^)] = 0.038
*wR*(*F* ^2^) = 0.093
*S* = 1.00
2412 reflections
145 parameters
H-atom parameters constrained
Δρ_max_ = 0.21 e Å^−3^
Δρ_min_ = −0.25 e Å^−3^

### 

Data collection: *RAPID-AUTO* (Rigaku, 2004 ▶); cell refinement: *RAPID-AUTO*; data reduction: *RAPID-AUTO*; program(s) used to solve structure: *SHELXS97* (Sheldrick, 2008 ▶); program(s) used to refine structure: *SHELXL97* (Sheldrick, 2008 ▶); molecular graphics: *XP* in *SHELXTL* (Sheldrick, 2008 ▶); software used to prepare material for publication: *SHELXL97*.

## Supplementary Material

Crystal structure: contains datablocks global, I. DOI: 10.1107/S1600536809039051/ci2926sup1.cif

Structure factors: contains datablocks I. DOI: 10.1107/S1600536809039051/ci2926Isup2.hkl

Additional supplementary materials:  crystallographic information; 3D view; checkCIF report

## Figures and Tables

**Table 1:** Hydrogen-bond geometry (Å, °)

*D*—H⋯*A*	*D*—H	H⋯*A*	*D*⋯*A*	*D*—H⋯*A*
C10—H10*B*⋯N2^i^	0.99	2.50	3.4890 (14)	173
C7—H7⋯*Cg*1^ii^	0.95	2.66	3.5220 (1)	151
C13—H13⋯*Cg*2^iii^	0.95	2.80	3.5660 (3)	139

Symmetry codes: (i) 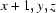; (ii) 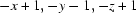; (iii) 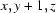. *Cg*1 and *Cg*2 are the centroids of the C11–C16 and C4–C9 rings, respectively.

**Fig. 1.**
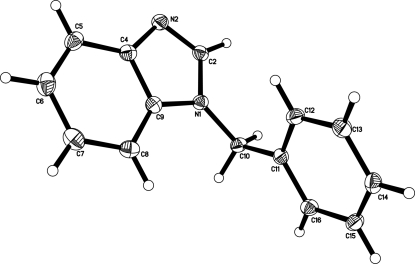
The molecular structure of the title compound, showing 50% probability displacement ellipsoids and the atomic numbering.

## supplementary crystallographic information

### Comment

Benzimidazole derivatives are a class of important compounds which exhibit antimicrobial activity (Ansari & Lal, 2009). Here, we report the crystal structure of the title compound.

Bond lengths and angles in the title molecule are normal. The benzimidazole ring system is planar, with a maximum deviation of 0.035 (2)Å for atom C4. The imidazole ring and benzene ring in benzyl group are almost mutually perpendicular, with a dihedral angle of 85.77 (4)% (Fig. 1). The crystal packing is stabilized by C—H···N hydrogen bonds and C—H···π interactions (*Cg*1 is the centroid of the C11-C16 ring and *Cg*2 is the centroid of the C4—C9 ring) (Table 1).

### Experimental

The title compound was synthesized according to the method reported in the literature (Hayat *et al.*, 2001). Colourless single crystals suitable for X-ray diffraction were obtained by slow evaporation of a methanol solution.

### Refinement

All H atoms were placed in calculated positions, with C-H = 0.95 or 0.99 Å, and refined using a riding model, with *U*_iso_(H) = 1.2*U*_eq_(C).

### Crystal data

**Table:**

C_14_H_12_N_2_	*F*(000) = 440
*M**_r_* = 208.26	*D*_x_ = 1.308 Mg m^−^^3^
Monoclinic, *P*2_1_/*n*	Mo *K*α radiation, λ = 0.71073 Å
Hall symbol: -P 2yn	Cell parameters from 3307 reflections
*a* = 6.2265 (10) Å	θ = 3.2–27.5°
*b* = 8.1740 (13) Å	µ = 0.08 mm^−^^1^
*c* = 20.975 (4) Å	*T* = 93 K
β = 97.839 (2)°	Block, colourless
*V* = 1057.5 (3) Å^3^	0.57 × 0.50 × 0.37 mm
*Z* = 4	

### Data collection

**Table:**

Rigaku SPIDER diffractometer	2198 reflections with *I* > 2σ(*I*)
Radiation source: Rotating Anode	*R*_int_ = 0.022
graphite	θ_max_ = 27.5°, θ_min_ = 3.2°
ω scans	*h* = −8→8
8358 measured reflections	*k* = −10→10
2412 independent reflections	*l* = −27→27

### Refinement

**Table:**

Refinement on *F*^2^	Primary atom site location: structure-invariant direct methods
Least-squares matrix: full	Secondary atom site location: difference Fourier map
*R*[*F*^2^ > 2σ(*F*^2^)] = 0.038	Hydrogen site location: inferred from neighbouring sites
*wR*(*F*^2^) = 0.093	H-atom parameters constrained
*S* = 1.00	*w* = 1/[σ^2^(*F*_o_^2^) + (0.0482*P*)^2^ + 0.296*P*] where *P* = (*F*_o_^2^ + 2*F*_c_^2^)/3
2412 reflections	(Δ/σ)_max_ = 0.001
145 parameters	Δρ_max_ = 0.21 e Å^−^^3^
0 restraints	Δρ_min_ = −0.25 e Å^−^^3^

### Special details

**Table:**

Geometry. All e.s.d.'s (except the e.s.d. in the dihedral angle between two l.s. planes) are estimated using the full covariance matrix. The cell e.s.d.'s are taken into account individually in the estimation of e.s.d.'s in distances, angles and torsion angles; correlations between e.s.d.'s in cell parameters are only used when they are defined by crystal symmetry. An approximate (isotropic) treatment of cell e.s.d.'s is used for estimating e.s.d.'s involving l.s. planes.
Refinement. Refinement of *F*^2^ against ALL reflections. The weighted *R*-factor *wR* and goodness of fit *S* are based on *F*^2^, conventional *R*-factors *R* are based on *F*, with *F* set to zero for negative *F*^2^. The threshold expression of *F*^2^ > σ(*F*^2^) is used only for calculating *R*-factors(gt) *etc*. and is not relevant to the choice of reflections for refinement. *R*-factors based on *F*^2^ are statistically about twice as large as those based on *F*, and *R*- factors based on ALL data will be even larger.

### Fractional atomic coordinates and isotropic or equivalent isotropic displacement parameters (Å^2^)

**Table:**

	*x*	*y*	*z*	*U*_iso_*/*U*_eq_	
N1	0.41468 (13)	0.11825 (10)	0.58340 (4)	0.0151 (2)	
N2	0.08985 (14)	0.00680 (11)	0.59213 (4)	0.0184 (2)	
C2	0.21067 (16)	0.08189 (12)	0.55458 (5)	0.0164 (2)	
H2	0.1602	0.1086	0.5110	0.020*	
C4	0.22460 (16)	−0.00841 (12)	0.65071 (5)	0.0164 (2)	
C5	0.18809 (18)	−0.08552 (13)	0.70781 (5)	0.0206 (2)	
H5	0.0516	−0.1329	0.7122	0.025*	
C6	0.35775 (19)	−0.09021 (14)	0.75762 (5)	0.0234 (3)	
H6	0.3374	−0.1434	0.7966	0.028*	
C7	0.55957 (19)	−0.01845 (14)	0.75220 (5)	0.0230 (3)	
H7	0.6720	−0.0241	0.7876	0.028*	
C8	0.59841 (17)	0.06012 (13)	0.69652 (5)	0.0190 (2)	
H8	0.7338	0.1103	0.6929	0.023*	
C9	0.42797 (16)	0.06166 (12)	0.64598 (5)	0.0153 (2)	
C10	0.58686 (16)	0.19667 (13)	0.55403 (5)	0.0163 (2)	
H10A	0.5488	0.1927	0.5067	0.020*	
H10B	0.7223	0.1334	0.5653	0.020*	
C11	0.62877 (16)	0.37280 (13)	0.57428 (5)	0.0148 (2)	
C12	0.47606 (16)	0.46721 (13)	0.59998 (5)	0.0175 (2)	
H12	0.3414	0.4201	0.6066	0.021*	
C13	0.51869 (18)	0.63021 (13)	0.61606 (5)	0.0198 (2)	
H13	0.4138	0.6936	0.6339	0.024*	
C14	0.71437 (17)	0.70020 (13)	0.60598 (5)	0.0187 (2)	
H14	0.7429	0.8119	0.6165	0.022*	
C15	0.86811 (17)	0.60677 (13)	0.58054 (5)	0.0185 (2)	
H15	1.0023	0.6544	0.5738	0.022*	
C16	0.82598 (16)	0.44380 (13)	0.56497 (5)	0.0171 (2)	
H16	0.9322	0.3801	0.5478	0.021*	

### Atomic displacement parameters (Å^2^)

**Table:**

	*U*^11^	*U*^22^	*U*^33^	*U*^12^	*U*^13^	*U*^23^
N1	0.0142 (4)	0.0141 (4)	0.0168 (4)	−0.0009 (3)	0.0017 (3)	−0.0012 (3)
N2	0.0160 (4)	0.0162 (4)	0.0226 (5)	−0.0005 (3)	0.0016 (3)	0.0004 (4)
C2	0.0155 (5)	0.0136 (5)	0.0195 (5)	0.0007 (4)	−0.0002 (4)	−0.0013 (4)
C4	0.0162 (5)	0.0131 (5)	0.0201 (5)	0.0014 (4)	0.0030 (4)	−0.0028 (4)
C5	0.0211 (5)	0.0181 (5)	0.0239 (5)	−0.0002 (4)	0.0084 (4)	−0.0008 (4)
C6	0.0305 (6)	0.0229 (6)	0.0178 (5)	0.0026 (5)	0.0074 (4)	0.0005 (4)
C7	0.0256 (6)	0.0248 (6)	0.0176 (5)	0.0029 (5)	−0.0006 (4)	−0.0033 (4)
C8	0.0171 (5)	0.0193 (5)	0.0205 (5)	−0.0003 (4)	0.0015 (4)	−0.0040 (4)
C9	0.0174 (5)	0.0119 (5)	0.0172 (5)	0.0017 (4)	0.0037 (4)	−0.0028 (4)
C10	0.0140 (5)	0.0167 (5)	0.0188 (5)	−0.0008 (4)	0.0043 (4)	−0.0016 (4)
C11	0.0154 (5)	0.0159 (5)	0.0129 (5)	0.0001 (4)	0.0009 (4)	0.0012 (4)
C12	0.0143 (5)	0.0187 (5)	0.0201 (5)	−0.0008 (4)	0.0039 (4)	0.0000 (4)
C13	0.0198 (5)	0.0173 (5)	0.0227 (5)	0.0031 (4)	0.0048 (4)	−0.0009 (4)
C14	0.0223 (5)	0.0143 (5)	0.0189 (5)	−0.0009 (4)	0.0010 (4)	0.0005 (4)
C15	0.0165 (5)	0.0198 (5)	0.0192 (5)	−0.0036 (4)	0.0027 (4)	0.0019 (4)
C16	0.0154 (5)	0.0196 (5)	0.0170 (5)	0.0009 (4)	0.0044 (4)	−0.0001 (4)

### Geometric parameters (Å, °)

**Table:**

N1—C2	1.3630 (13)	C8—H8	0.95
N1—C9	1.3835 (13)	C10—C11	1.5137 (15)
N1—C10	1.4560 (13)	C10—H10A	0.99
N2—C2	1.3132 (14)	C10—H10B	0.99
N2—C4	1.3956 (13)	C11—C12	1.3892 (14)
C2—H2	0.95	C11—C16	1.3958 (14)
C4—C5	1.3992 (15)	C12—C13	1.3910 (15)
C4—C9	1.4055 (14)	C12—H12	0.95
C5—C6	1.3812 (16)	C13—C14	1.3881 (15)
C5—H5	0.95	C13—H13	0.95
C6—C7	1.4053 (17)	C14—C15	1.3870 (15)
C6—H6	0.95	C14—H14	0.95
C7—C8	1.3826 (16)	C15—C16	1.3881 (16)
C7—H7	0.95	C15—H15	0.95
C8—C9	1.3940 (14)	C16—H16	0.95
			
C2—N1—C9	106.23 (9)	N1—C10—C11	114.12 (8)
C2—N1—C10	127.14 (9)	N1—C10—H10A	108.7
C9—N1—C10	126.61 (8)	C11—C10—H10A	108.7
C2—N2—C4	104.17 (9)	N1—C10—H10B	108.7
N2—C2—N1	114.29 (9)	C11—C10—H10B	108.7
N2—C2—H2	122.9	H10A—C10—H10B	107.6
N1—C2—H2	122.9	C12—C11—C16	119.02 (10)
N2—C4—C5	130.19 (10)	C12—C11—C10	122.43 (9)
N2—C4—C9	109.93 (9)	C16—C11—C10	118.52 (9)
C5—C4—C9	119.80 (10)	C11—C12—C13	120.53 (10)
C6—C5—C4	117.59 (10)	C11—C12—H12	119.7
C6—C5—H5	121.2	C13—C12—H12	119.7
C4—C5—H5	121.2	C14—C13—C12	120.02 (10)
C5—C6—C7	121.82 (10)	C14—C13—H13	120.0
C5—C6—H6	119.1	C12—C13—H13	120.0
C7—C6—H6	119.1	C15—C14—C13	119.86 (10)
C8—C7—C6	121.57 (10)	C15—C14—H14	120.1
C8—C7—H7	119.2	C13—C14—H14	120.1
C6—C7—H7	119.2	C14—C15—C16	120.05 (10)
C7—C8—C9	116.34 (10)	C14—C15—H15	120.0
C7—C8—H8	121.8	C16—C15—H15	120.0
C9—C8—H8	121.8	C15—C16—C11	120.51 (10)
N1—C9—C8	131.71 (10)	C15—C16—H16	119.7
N1—C9—C4	105.38 (9)	C11—C16—H16	119.7
C8—C9—C4	122.85 (10)		
			
C4—N2—C2—N1	−0.14 (12)	N2—C4—C9—N1	−0.51 (11)
C9—N1—C2—N2	−0.17 (12)	C5—C4—C9—N1	176.66 (9)
C10—N1—C2—N2	178.24 (9)	N2—C4—C9—C8	−177.95 (9)
C2—N2—C4—C5	−176.38 (11)	C5—C4—C9—C8	−0.78 (15)
C2—N2—C4—C9	0.40 (11)	C2—N1—C10—C11	105.93 (11)
N2—C4—C5—C6	176.02 (10)	C9—N1—C10—C11	−75.97 (12)
C9—C4—C5—C6	−0.49 (15)	N1—C10—C11—C12	−20.55 (13)
C4—C5—C6—C7	0.97 (16)	N1—C10—C11—C16	161.34 (9)
C5—C6—C7—C8	−0.20 (17)	C16—C11—C12—C13	0.16 (15)
C6—C7—C8—C9	−1.03 (16)	C10—C11—C12—C13	−177.95 (10)
C2—N1—C9—C8	177.52 (11)	C11—C12—C13—C14	0.52 (16)
C10—N1—C9—C8	−0.91 (17)	C12—C13—C14—C15	−0.72 (16)
C2—N1—C9—C4	0.41 (11)	C13—C14—C15—C16	0.24 (16)
C10—N1—C9—C4	−178.02 (9)	C14—C15—C16—C11	0.45 (15)
C7—C8—C9—N1	−175.17 (10)	C12—C11—C16—C15	−0.64 (15)
C7—C8—C9—C4	1.52 (15)	C10—C11—C16—C15	177.54 (9)

### Hydrogen-bond geometry (Å, °)

**Table:**

*D*—H···*A*	*D*—H	H···*A*	*D*···*A*	*D*—H···*A*
C10—H10B···N2^i^	0.99	2.50	3.4890 (14)	173
C7—H7···Cg1^ii^	0.95	2.66	3.5220 (1)	151
C13—H13···Cg2^iii^	0.95	2.80	3.5660 (3)	139

Symmetry codes: (i) *x*+1, *y*, *z*; (ii) −*x*+1, −*y*−1, −*z*+1; (iii) *x*, *y*+1, *z*.
